# Retrospective Study of Seven Cases with Acute Fatty Liver of Pregnancy

**DOI:** 10.1155/2013/730569

**Published:** 2013-06-27

**Authors:** Suchi Dwivedi, Ma Runmei

**Affiliations:** Department of Obstetrics & Gynaecology, First Affiliated Hospital of Kunming Medical University, Kunming, Yunnan 650032, China

## Abstract

*Objectives*. Our aim is to explore the clinical outcome of patients with acute fatty liver of pregnancy (AFLP), and evaluate the effect of early diagnosis and treatment. *Methods*. Seven patients who were diagnosed with AFLP were retrospectively analyzed from February 2005 to January 2013. The clinical records of the patients with AFLP were reviewed for clinical features, laboratory examinations, and maternal and perinatal prognosis. Routine laboratory evaluation revealed hyperbilirubinemia, moderately elevated liver transaminase, but negative serum hepatitis virus in each patient. For additional evidence, 126 cases of AFLP were reviewed retrospectively from original articles researched in A Medline-based English and Chinese Knowledge Infrastructure between the same periods. *Results*. The initial symptoms of all the 7 cases with AFLP were gastrointestinal symptoms; anorexia, nausea, vomiting, and progressive jaundice. Complications revealed with renal insufficiency in all 7 patients. Hepatic failure, MODS, hypoglycemia and DIC were seen in 4 patients (57.1%). Hemorrhagic shock, ARDS, and hepatic encephalopathy were seen in 3 patients (42.8%). There was only one case of maternal death (14.2%), three cases of perinatal death (30%) and one postnatal death (10%). *Conclusion*. AFLP occurs in late pregnancy is a rare clinical syndrome occurs at about 36 weeks of gestation. Early diagnosis and prompt termination of pregnancy is the key of management with multidisciplinary collaboration, comprehensive treatment and effective prevention are helpful to improve prognosis of the cases with AFLP and perinatal death.

## 1. Introduction

Acute fatty liver of pregnancy (AFLP) is a rare and potentially life-threatening complication which tends to manifest in the third trimester of pregnancy/early postpartum period. The condition occurs more commonly in primigravida, twin pregnancy, and pregnancies carrying a male fetus. Maternal mortality is now estimated to be 12.5%–18%, with a neonatal mortality rate of 7%–66% [1].

The incidence of AFLP is 1 in 7,000 to 1 in 16,000 pregnancies [2]. A prospective UK-based research study, involving 229 centers identified 57 confirmed cases in a total of 1,132,964 pregnancies, giving an incidence of 5 in 100,000 pregnancies [3]. 74% of cases were identified at a median gestation age of 36 weeks, with 60% of cases delivered within 24 h of diagnosis [3]. The caesarean section rate was 74%. The mortality due to AFLP has declined in recent years. Though, it is important to further lower the mortality rate by early diagnosis and prompt treatment of this disease. Some cases of AFLP have been analyzed retrospectively for improvement of comprehensive knowledge. The aim of the present study is to explore the clinical outcome of patients with AFLP, evaluate the effect of early diagnosis and treatment, and analyze the influence of the mode of delivery on <maternal-perinatal mortalities.

The exact etiology of AFLP is unknown, but evidence points to hepatic damage caused by defects in the activity of long chain 3-hydroxyacyl coenzyme A dehydrogenase (LCHAD) [4]. Toxins produced from aberrant fatty acid oxidation may play a role in the development of AFLP. Moreover, the interaction between an LCHAD activity-deficient female and similarly deficient fetus may also lead to the progression to AFLP and could explain the rarity of the disease [4].

The pathophysiology of AFLP is related to the coagulopathy common in other types of acute liver failure. Patients typically present with unremitting nausea, vomiting, weight loss, and abdominal pain. Jaundice usually develops after several days. Abnormal laboratory findings include moderate elevations of aminotransferase levels (300 to 500 U/L), bilirubin ranging from 3 to 25 mg/dL, markedly elevated alkaline phosphatase levels (normal, 42 to 98 U/L), and leukocytosis. Abnormal coagulation parameters are found in association with secondary disseminated intravascular coagulation. Profound hypoglycemia occurs as liver dysfunction progresses.

Acute fatty liver of pregnancy is characterized by hepatic microvesicular steatosis and known to be associated with defective fatty acid oxidation in fetus. Bleeding, disseminated intravascular coagulation (DIC), and renal insufficiency are one of most common complications. Early diagnosis and better management with prompt termination of pregnancy have improved the prognosis with better maternal and fetal outcomes [5].

AFLP occurring late in pregnancy is characterized by the signs of acute hepatic failure with nonspecific symptoms such as nausea, vomiting, fatigue, thirst, headache, jaundice, and altered mental status. If untreated, AFLP can lead to coagulopathy, fulminant hepatic failure, multiple organ dysfunction, and death. AFLP is associated with raised bilirubin and transaminase levels; other possible features include hyperuricemia and thrombocytopenia. The diagnosis of AFLP can be challenging. So, the best approach to any pregnant women with liver dysfunction is to quickly rule out other, more likely, causes. Although the gold standard for diagnosis is liver biopsy, this is rarely necessary as it can cause complications in the presence of coagulopathy. The Swansea diagnostic criteria are an alternative to liver biopsy. The disease progression may lead to renal failure, clotting disorders, and hypoglycemia. Because of the potential for rapid progression to coma and death, AFLP is considered as an obstetrics emergency. 

It is important to consider prompt delivery once the diagnosis has been evident for AFLP and any serious maternal biochemical or hematological abnormalities have been corrected. Delivery is the definitive treatment for AFLP. Inducement may suffice, but cesarean section may be required. AFLP patients generally improve soon after delivery unless hepatic encephalopathy has developed. Most of the patients stabilize after delivery of fetus. Moderately or severely affected patients (those presenting with encephalopathy, deep jaundice, or prothrombin times less than 40% of control) or patients with any extrahepatic complication should be attended to in the intensive care unit. Affected women and their offspring should be screened for disorders of *β*-fatty acid oxidation as AFLP occurs more commonly in heterozygous mothers who are carrying fetuses that are homozygous for these disorders [6].

## 2. Patients and Methods

With approval from the department of obstetrics and gynecology, we identified and reviewed the medical records of all patients with the diagnosis of AFLP managed at the first affiliated hospital of Kunming Medical University between February 2005 and January 2013.

### 2.1. Patients

A total of 7 patients with AFLP, were admitted to this study. All patients were admitted to the obstetrics emergency and medical intensive care unit (ICU) for collaborative management with intensivists and medical subspecialists until the AFLP disease process was deemed to be well into its recovery phase.

The clinical records of the patients with AFLP were reviewed for clinical features, laboratory examinations, and complications and maternal and perinatal prognosis. The mean maternal age at diagnosis was 26 years (range 21–32 years). The mean gestational age at AFLP diagnosis was 36 weeks (range 32–38 weeks). Six patients were primigravida and one patient was multigravida. There were four single and three twin pregnancies. Fetal sex included eight males and two females.

### 2.2. Diagnosis

The diagnosis of AFLP was made on the basis of clinical and laboratory criteria as follows: (i) patients with symptoms of nausea, vomiting, epigastric pain, polydipsia/polyuria, jaundice, and abnormal liver function in the third trimester of pregnancy; (ii) characteristic laboratory examination; (iii) ultrasound imaging showing fatty liver. The diagnosis of AFLP was supported by a combination of clinical and laboratory features reflecting hepatorenal compromise, encephalopathy, and a consumptive coagulopathy. All cases conformed to the diagnostic criteria mentioned above, except for liver biopsy. Because of their severe conditions, prolonged prothrombin times, reduced platelet counts, and/or the patients' refusal, patients did not receive liver biopsy. All patients exhibited six or more of the Swansea criteria to objectively confirm the diagnosis of AFLP: vomiting, abdominal pain, polydipsia/polyuria, encephalopathy, elevated serum bilirubin level, elevated uric acid, hypoglycemia, leukocytosis, elevated transaminases, ascites or bright liver on ultrasound scan, elevated ammonia, renal impairment, coagulopathy, and microvesicular steatosis on liver biopsy in addition to metabolic acidosis and occasionally biochemical pancreatitis.

Furthermore, an additional research of 126 cases with AFLP was reviewed retrospectively from the Medline-based Knowledge Infrastructure search on original published papers (English and Chinese) between February 2005 and January 2013. The contents of this finding include mean age, gestational age, modes of delivery, complications, and maternal-perinatal outcomes. These contents are emblematized in Section 4.

## 3. Results

Seven patients were identified for retrospective study. All 7 patients diagnosed with AFLP were presented with clinical features of nausea, vomiting, epigastric pain, polydipsia/polyuria, jaundice, and abnormal liver functions in the third trimester of pregnancy. Persistent vomiting, epigastric pain in 6 out of 7 (85.7%), and jaundice in all patients were the cardinal features for diagnostics of AFLP. The clinical manifestations of acute fatty liver of pregnancy are listed below in Table 1.

### 3.1. Pregnancy Outcome

The mean gestational age was 36 weeks (range 32–38 weeks). Six patients were primigravida and one patient was multigravida. There were four single and three twin pregnancies. The interval between onset of first symptoms and the delivery of fetus was 8 days (range, 3–14 days). Among 7 patients, five undergone cesarean section (71.4%) and two patients delivered vaginally (28.5%). Fetal sex included eight males (80%) and two females (20%). Three intrauterine fetal deaths (30%) and one postnatal death (10%) occurred. All six neonates which survived successfully, were noted one-minute Apgar score of 4 (range, 3–5) and five-minute Apgar score was 7. The outcomes of pregnancy with age, gravida, and gestational age at the onset of disease are mentioned below in Table 2.

The abnormal laboratory results of the seven cases were as follows: elevated hepatic aminotransferase levels (alanine aminotransferase and aspartate aminotransferase), total bilirubin, direct bilirubin, blood urea nitrogen, creatinine, leukocytosis, decreased platelet counts, and prolonged prothrombin time. Some patients also had hypoglycemia. Elevated total serum bilirubin concentration ranged from 178.5 to 536. 1 *μ*mol/L. Hepatitis screening was negative in all patients. All cases were reported, with varying degrees of thrombocytopenia, prolonged PT, and prolonged PTT, as dysfunction of blood coagulations, which were attributable to DIC or hepatic inadequacy. The laboratory results of blood biochemistry and hematology found in acute phase of AFLP—shortly before delivery—are mentioned in Table 3. 

All the 7 patients in this study underwent ultrasound examinations. All the images displayed a bright hepatic echo pattern, indicating fatty liver disease. None of the patients underwent CT; histological examination of the tissue from placenta stained with oil red O was consistent with AFLP and revealed abnormal placenta. Because of their serious conditions, prolonged prothrombin times, decreased platelet counts, highest bleeding tendencies, and/or the patient's refusal, liver biopsy was not done.

### 3.2. Maternal Morbidity and Mortality

The most common clinical complications were renal insufficiency in all patients; hepatic failure and multiple organ dysfunction syndrome (MODS), hypoglycemia, disseminated intravascular coagulation (DIC) failure in 4 patients (57.1%), hemorrhagic shock, ARDS, and hepatic encephalopathy were seen in 3 patients (42.8%). Two cases had ascites and metabolic acidosis (28.5%). There was only one maternal death which occurred (14.2%). Six of the mothers improved after their pregnancies were terminated and were discharged at an average of 12 days; seventh case worsened after cesarean section, exhibiting persistent jaundice, ascites, oliguria, hepatic encephalopathy, and bleeding tendencies, and were treated in ICU with aggressive supportive therapy, such as correction of liver function, therapy to affect jaundice reduction, therapy to diminish liver enzymes, correction for coagulation dysfunction, and antibiotic therapy. Six cases were discharged in stable condition and one case ultimately died. The women died 15 days after admission secondary to multiorgan failure; as she was complicated by acute respiratory distress syndrome, hepatic failure, renal insufficiency, sepsis, disseminated intravascular coagulation (DIC), and encephalopathy. She was also associated to hemorrhagic shock due to intraabdominal bleeding and upper gastrointestinal hemorrhage; the condition quickly deteriorated after cesarean section. The complications associated with AFLP are presented in Table 4 and the total duration of hospital stay in Table 5.

In our data analysis, five pregnancies were terminated by cesarean section, and there were only two vaginal deliveries. The data available for comparison are mentioned in Table 2. Therefore, to explore the choice of mode of delivery, seven cases from our hospital and overall 126 cases from the Medline-based Knowledge Infrastructure search on original published papers (English and Chinese) between the same periods were analyzed retrospectively. Of the 126 patients, 109 (86.5%) pregnancies were terminated by cesarean section, and of those cases 14 (12.8%) died. Seventeen and (13.4%) patients delivered vaginally, resulting in 6 (35.2%) deaths. The mortality rate of the mothers who underwent cesarean Section 12.8% was lower than those who delivered vaginally 35.2%.

Furthermore, there were 12 (8.9%) perinatal deaths from cesarean section delivery and 7 (28%) perinatal deaths from vaginal delivery. In addition, an overall summary on the 126 patients including the clinical complications, management, pregnancy outcome are present in Table 6.

## 4. Discussion

AFLP is rare, late-gestational, potentially life-threatening complication of pregnancy which occurs at mean gestational age of 36 weeks (range 32–38 weeks). It is reported that being primigravida, having had multiple pregnancies, carrying a male fetus, and experiencing preeclampsia are the high-risk factors for AFLP [1]. Preeclampsia occurs in approximately 50% of AFLP cases, and 15% of cases are associated with multiple pregnancies [7]. Our data confirms the similar situation mentioned above. Since AFLP shows atypical presentation with abrupt evolution of several complications resulting in multiple organ failure, it needs emergency referral and prompt treatment. Summarizing the data from 126 cases, AFLP should be highly suspected when the following clinical conditions occur: first (i) gastrointestinal symptoms, which include nausea, vomiting, vague abdominal pain, polydipsia/polyuria, and persistent jaundice appearing in late pregnancy without obvious reason; second (ii) abnormal liver function occurring in late pregnancy, when other hepatic diseases have been excluded; laboratory examination showing elevated leukocytes, total bilirubin, especially direct bilirubin, prothrombin time, hepatic aminotransferase levels (ALT, AST), and decreased platelets; and third (iii) pregnancy complicated rapidly with renal insufficiency, hepatic inadequacy, coagulopathy, hypoglycemia, and hepatic encephalopathy, along with multiple organ dysfunction. These prompt complicating manifestations tend to worsen the conditions seeking urgent medical attention in obstetrics emergency and medical intensive care unit (ICU) for collaborative management.

Both ultrasound and CT scan are the most common methods for clinical diagnosis of fatty liver but the specificity and sensitivity of these studies are insufficient to provide a confirmatory diagnosis of AFLP, with high possibility of false negative results [8]. For safety and convenience, ultrasound is seen as the choice of method in screening for fatty liver. In our study, the rate of positive AFLP diagnosis by ultrasound was 100%. In recent years, noninvasive methods have often been used for early diagnosis of AFLP. Liver biopsy is the gold standard for confirmatory diagnosis of fatty liver. However, it is restricted to clinical use in patients whose conditions are complicated by coagulopathy and bleeding tendencies as a result of prolonged prothrombin times, reduced platelet counts. Liver biopsy needs to be performed early in the preliminary diagnosis, before the occurrence of several complications. Therefore, we suggest the routine use of ultrasound or CT for early diagnosis of AFLP. 

According to the Swansea [9], criteria for diagnosis of AFLP include six or more of the following features in the absence of another explanation: vomiting, abdominal pain, polydipsia/polyuria, encephalopathy, elevated bilirubin (>14 *μ*mol/L), leukocytosis (>11 × 10^9^/L), elevated uric acid (>340 *μ*mol/L), hypoglycemia (<4 mmol/L), ascites on ultrasound scan, increase in the level of transaminases (aspartate aminotransferase or alanine aminotransferase > 42 IU/L), renal impairment (creatinine > 150 *μ*mol/L), elevated ammonia (>47 *μ*mol/L), coagulopathy (prothrombin time > 14 sec or activated partial thromboplastin time > 34 sec), and microvesicular steatosis on liver biopsy. The most striking feature of this syndrome is a high level of bilirubin associated with moderate increases of transaminases. The platelet count may be decreased with or without other signs of disseminated intravascular coagulation (DIC). Nearly 90% of the women with AFLP had an abdominal ultrasound examination. Classical features of ascites or bright liver were only seen in a quarter of these. This observation is reflected in other studies which report that hepatic ultrasound is not sufficiently sensitive or specific to make a definite diagnosis.

The highly suspicious or confirmed cases of AFLP must be treated as obstetrics emergency, with the prompt termination of pregnancy. Early diagnosis and intervention can result in better maternal and fetal outcomes. The management of AFLP requires maternal stabilization following delivery and supportive care. In our data, all pregnancies were terminated within 24 hours of highly suspected or diagnosed AFLP, and the outcomes were good. Therefore, timely termination of pregnancy is crucial in the treatment of AFLP. If vaginal delivery cannot be achieved quickly, cesarean section is the preferred method, as it is beneficial to stop the progress of the patient's condition and to shorten labor as much as possible. In conclusion, there is no specific and selective consideration mode of pregnancy termination. In above mentioned data, of the 126 patients, 109 (86.5%) pregnancies were terminated by cesarean section and of those cases 14 patients died. Seventeen (13.4%) patients delivered vaginally resulting in 6 deaths. The mortality rate of the mothers who underwent cesarean Section 12.8% was lower than those who delivered vaginally 35.2%. There were 12 (8.9%) perinatal deaths delivered by cesarean section and 7 (28%) perinatal deaths from vaginal delivery. So, analyzing these data prompt delivery after confirmed diagnosis is essential for better pregnancy outcome and lesser maternal and fetal mortality.

Delivery is the definitive treatment for AFLP. Inducement may suffice, but cesarean section may be required. AFLP patients generally improve soon after delivery unless hepatic encephalopathy has developed. All patients should be hospitalized as soon as a diagnosis of AFLP is suspected. Moderately or severely affected patients (those presenting with encephalopathy, deep jaundice, or prothrombin times less than 40% of control) or patients with any extrahepatic complication should be attended to in the intensive care unit. Glucose infusions should be maintained until a full metabolic recovery is achieved due to the risk of sudden hypoglycemia, which can occur at any time, even during clinical recovery. Most patients require platelets and fresh frozen plasma infusions. Prothrombin time and blood glucose levels should be monitored daily or more often. Other supportive care is provided based upon clinical symptoms.

Patients with severe hepatic dysfunction are best treated in an ICU setting before and after delivery. Medical management of patients with AFLP is supportive. Blood sugar levels should be monitored and severe coagulation disorders treated with platelets and fresh frozen plasma transfusions. Initial treatment involves supportive management with intravenous infusion, intravenous glucose, fresh frozen plasma, and packed red blood cells for patients with coagulation dysfunction to reduce blood loss and cryoprecipitate to correct DIC. Once the patient is stabilized, further course of management includes the appropriate mode for delivery. This may occurs by vaginal delivery, while in cases of severe bleeding and compromise status of mother, caesarian section may be done [10]. When coagulation dysfunction exists, patients have an additional degree of risk with cesarean section; in these cases, a longitudinal incision is beneficial to reduce bleeding. Therefore, we suggest that cesarean section is the method of choice to terminate pregnancy, if vaginal delivery cannot be performed promptly. Because the probability of postpartum hemorrhage is high, hysterectomy and uterine artery embolization should be considered at the time of pregnancy termination. The clinical conditions deteriorate rapidly and progress to multiorgan dysfunctions, if there is delay in the diagnosis and the prompt termination of pregnancy. 

Improvement usually begins with delivery. Patients with severe hepatic injury remain at risk for respiratory failure, renal failure, GI bleeding, and nephrogenic diabetes insipidus and should be closely monitored during the immediate postpartum period. The rare patient who progresses to fulminant hepatic failure can be treated by liver transplantation. Surviving patients generally recover with no hepatic sequel. Further pregnancies are often uncomplicated but remain at risk for recurrent AFLP.

Systemic complications of AFLP are due to fulminant hepatic failure and include encephalopathy, and renal insufficiency, coagulopathy, sepsis, hypoglycemia, and gastrointestinal hemorrhage. Other systemic complicating effects include acute respiratory distress syndrome sometimes requiring assisted ventilation, ascites, [8] and upper gastrointestinal bleeding from gastric ulceration, and Mallory-Weiss syndrome [11]. Maternal deaths occurs due to hemorrhage, gastrointestinal bleeding, sepsis, aspiration, pancreatitis, renal failure [12].

## 5. Conclusion

AFLP is an uncommon, life-threatening complication of third trimester with variable presentation. It may occur rapidly and the progression is unpredictable. In cases of AFLP, a good maternal outcome is dependent on early diagnosis and immediate delivery. It is recommended that patients with nausea, vomiting, or epigastric pain, and persistent jaundice in the third trimester, with alter in liver function, should be suspected for the diagnosis of AFLP. The patients, who are critically ill at the time of clinical presentation, develop complications, or continue to deteriorate despite emergency delivery, and require collaborative management in the intensive care unit (ICU). Early diagnosis, prompt delivery, adequate supportive care, and a multidisciplinary approach are the key to a good outcome.

## Figures and Tables

**Table 1:** Clinical manifestations of acute fatty liver of pregnancy.

Symptoms	Patient I	Patient II	Patient III	Patient IV	Patient V	Patient VI	Patient VII
Vomiting	Yes	No	Yes	Yes	Yes	Yes	Yes
Epigastric pain	Yes	Yes	No	Yes	Yes	Yes	Yes
Jaundice	Yes	Yes	Yes	Yes	Yes	Yes	Yes
Polydipsia	Yes	Yes	No	Yes	No	No	Yes
Pruritis	Yes	No	No	Yes	No	Yes	No
Encephalopathy (stage 0–IV)	II	0	0	0	III	0	II

**Table 2:** Age, gravida, gestational age at onset of the disease, and outcome of pregnancy in 7 patients of acute fatty liver of pregnancy.

Patient Number	Age (years)	Gravida and parity	Onset of symptoms (weeks of pregnancy)	Interval between first symptoms and delivery (days)	Mode of delivery	Fetal sex	Fetal weight (g)	Apgar score at one and five minutes
I	27	G_1_P_0_	34	14	CS	M M	1800 1850	4–7 3–7
II	25	G_1_P_0_	36	3	V	F F	2110 2385	Stillborn Postnatal death
III	21	G_1_P_0_	36	10	V	M	2150	5–7
IV	22	G_1_P_0_	38	6	CS	M	2950	Stillborn
V	32	G_4_P_3_	32	9	CS	M M	1620 1420	5–7 4–7
VI	29	G_1_P_0_	38	5	CS	M	2600	Stillborn
VII	26	G_1_P_0_	38	8	CS	M	2510	3–7

V: vaginal delivery; CS: caesarean section.

**Table 3:** Results of blood biochemistry and hematology during acute phase of AFLP (shortly before delivery).

Findings	Mean ± S.D	Range
Aspartate aminotransferase (U/L)	177.5 ± 48.8	119–246
Alanine aminotransferase (U/L)	112.3 ± 39.4	60–178
Lactic dehydrogenase (U/L)	317.5 ± 73	234–428
Total bilirubin (*μ*mol/L)	327.4 ± 173	158.2–526
Direct bilirubin (*μ*mol/L)	182.6 ± 60.2	110–246
Blood urea nitrogen	24 ± 3.4	20–29
Prothrombin time (INR)	17 ± 7.3	6.2–26.4
Thrombin time	29.3 ± 3.8	25.5–35.4
Partial thromboplastin time (s)	52.4 ± 9	36.2–60.4
Fibrinogen (g/L)	0.9 ± 0.4	0.6–1.5
Total Protein (g/L)	40.3 ± 4.7	34–48
Creatinine (*μ*mol/L)	306 ± 83.7	218–441
Uric acid (*μ*mol/L)	671.8 ± 76.7	598–788
Glucose (mmol/L)	3 ± 1.5	0.9–5.1
WBC (×10^9^/L)	15.4 ± 3.8	10.3–18.8
Platelets (×10^9^/L)	39.5 ± 9.3	33–56

**Table 4:** Complications associated with AFLP.

Findings	Number of patients (*n* = 7)	Percentage (%)
Hypoglycemia	4	57.1
Acute renal insufficiency	7	100
DIC	4	57.1
Ascites	2	28.5
Liver failure	4	57.1
Hepatic encephalopathy	3	42.8
ARDS	3	42.8
Hemorrhagic shock	3	42.8
Pulmonary Edema	1	14.2
Sepsis	1	14.2
Metabolic acidosis	2	28.5
MODS	4	57.1
IGT	1	14.2
Maternal death	1	14.2

DIC: disseminated intravascular coagulation; ARDS: acute respiratory distress syndrome; MODS: multiple organ dysfunction syndrome; IGT: impaired glucose tolerance.

**Table 5:** Duration of stay in hospital and ICU.

Duration of stay	Patient I	Patient II	Patient III	Patient IV	Patient V	Patient VI	Patient VII
Hospital (days)	15	11	3	23	20	2	15
ICU (days)	14	1	2	20	17	2	2

**Table 6:** The overall summary of the 126 cases of AFLP.

Particulars	*n* = 7 cases	*n* = 126 cases	*P* value	*χ* ^2^ test
Maternal age (years)	26 years (Range, 21–32)	27 years (Range, 21–37)		
Mean gestational age (weeks)	36 weeks (Range, 32–38)	36 weeks (Range, 28–38)		
Primigravida	85.7 (6/7)	74.6 (94/126)	0.837	0.045
Multigravida	14.2 (1/7)	25.4 (32/126)		
Mode of delivery				
Cesarean section	71.4 (5/7)	86.5 (109/126)	0.579	0.308
Vaginal delivery	28.5 (2/7)	13.4 (17/126)		
Complications				
Hypoglycemia	57.1 (4/7)	41.2 (52/126)	0.664	0.189
Acute renal insufficiency	100 (7/7)	50.7 (64/126)	0.031	4.626
ARDS	42.8 (3/7)	40.4 (51/126)	1	0
Coagulopathy	57.1 (4/7)	78.5 (99/126)	0.392	0.732
Encephalopathy	42.8 (3/7)	46 (58/126)	1	0
MODS	57.1 (4/7)	30.1 (38/126)	0.281	1.16
Outcomes				
Maternal death				
Cesarean section	20 (1/5)	12.8 (14/109)	0.513	
Vaginal delivery	0 (0/2)	35.2 (6/17)	1	
Perinatal/postnatal death				
Cesarean section	28.5 (2/7)	8.9 (12/134)	0.144	
Vaginal delivery	66.6 (2/3)	28 (7/25)	0.234	

ARDS: Acute respiratory distress syndrome; MODS: Multiple organ dysfunction syndrome.
